# Stable isotopic signature of dissimilatory nitrate reduction is robust against enzyme mutation

**DOI:** 10.1073/pnas.2416002121

**Published:** 2024-11-22

**Authors:** Ciara K. Asamoto, Yeongjun Ryu, Kelly N. Eckartt, Julia Kelley-Kern, Lars E.P. Dietrich, Daniel M. Sigman, Sebastian H. Kopf

**Affiliations:** ^a^Department of Geological Sciences, University of Colorado Boulder, Boulder, CO 80309; ^b^Department of Geosciences, Princeton University, Princeton, NJ 08544; ^c^Department of Biological Sciences, Columbia University, New York, NY 10027

**Keywords:** enzyme, evolution, stable isotopes, nitrogen

## Abstract

The proportionality of oxygen-to-nitrogen isotope effects (^18^ε/^15^ε) is used as a key isotopic signature of nitrogen cycling processes in the environment. Dissimilatory nitrate reduction is observed to have an ^18^ε/^15^ε proportionality of ~0.9 in marine and ~0.6 in freshwater/terrestrial ecosystems. The origins of this difference are uncertain, with both geochemical and biological factors conceivably at play. One potential factor is variation in the isotope effect of nitrate reduction among different forms of the nitrate reductase enzyme. NarG nitrate reductases are observed to typically have an ^18^ε/^15^ε of ~0.9. However, a recent study uncovered an exception, with *Bacillus* NarG enzymes having an ^18^ε/^15^ε proportionality of ~0.6. This provides an opportunity to investigate genetic controls on ^18^ε/^15^ε. Furthermore, this atypical NarG signature also raises the question of whether intrinsic isotope signatures can evolve as the enzymes that produce them accumulate mutations through time. Here, we present data from site-directed mutagenesis experiments of key NarG residues, which suggest that the distinct *Bacillus* ^18^ε/^15^ε cannot be caused by single mutations alone and is potentially uncommon in nature. Variation in the intrinsic isotope effects of an enzyme through time may thus require more extensive evolutionary changes.

Many biogeochemical processes can lead to distinct isotopic differences in reactants and products that are quantified by fractionation factors (ε). In modern ecosystems, the proportionality of the fractionation of stable oxygen isotopes (^18^O, ^16^O) and nitrogen isotopes (^15^N, ^14^N)—the ^18^ε/^15^ε ratio—reflects the isotopic signature of different enzymes and thus processes within the nitrogen cycle (1). Enzymes NarG and NapA both catalyze the first step of denitrification, nitrate reduction to nitrite (NR) but have distinct ^18^ε/^15^ε of approximately 0.9 [0.85 to 1 (2–5)] and 0.6 [0.49 to 0.68 (2, 4, 6, 7)], respectively. These ^18^ε/^15^ε ratios match environmental observations in marine (~0.9) and freshwater systems (~0.6), generating ongoing discussion about the possible prevalence of NapA-mediated NR in freshwater systems versus other N-cycling processes as possible explanations for the lower ^18^ε/^15^ε of freshwater systems (1, 2). Furthermore, given the similarity of the active sites and functions of NapA and NarG nitrate reductases, the causes of differences in ^18^ε/^15^ε remain unknown. Prior research into why NapA enzymes fractionate nitrate differently than NarG using transition state modeling of the active site has yielded inconclusive results as to the mechanism for the lower ^18^ε/^15^ε in NapA, demonstrating our limited understanding of what controls isotopic fractionation at the enzyme level (8, 9). Previously, we uncovered an exception to the NapA vs. NarG isotopic distinction: *Bacillus vireti* and *Bacillus bataviensis* have ^18^ε/^15^ε ratios of ~0.6 (4), which is characteristic of NapA-mediated NR despite both species of bacteria having only a NarG reductase. This suggests that genetic variation in NarG enzymes can result in different ^18^ε/^15^ε signatures, presenting an opportunity for us to examine how genetic variability in enzymes may contribute to the ^18^ε/^15^ε signature.

Using an amino acid sequence alignment of all NarG enzymes with published ^18^ε/^15^ε ratios, we identified two key residues, positions 62 and 221 (*Escherichia coli* reference frame, PDB 1Q16), that could be responsible for the *Bacillus* ^18^ε/^15^ε anomaly (Figs. 1 and 2 *B* and *C*). To assess the importance of these residues for ^18^ε/^15^ε, we studied NarG of the gram-negative bacterium *Pseudomonas aeruginosa* PA14. A PA14 strain lacking NapA (PA14 ∆*napA*) has the typical ^18^ε/^15^ε of ~0.9 (4). Using PA14 ∆*napA* as the parent, we constructed the three mutant strains NarG Y62H, NarG C221A, and NarG Y62H C221A (double mutant). Position 62 in NarG is near the enzyme’s iron–sulfur cluster (FS0) and at the interface with the electron-transferring subunit NarH and, thus, may influence rates of electron transfer so as to impact isotope fractionation (Fig. 2*C*.). The mutation at position 221 is shared by almost all nonproteobacterial strains and is located in the active site of the enzyme, directly next to the Mo-coordinating aspartic acid residue (Fig. 2*B*.). Additionally, to expand the available ^18^ε/^15^ε data to capture NarG isotope signatures from outside *Bacillus* and proteobacterial taxa, we cultured and collected ^18^ε/^15^ε data for *Staphylococcus carnosus* and *Corynebacterium marinum*, two species of bacteria that share many of the sequence changes in the *Bacillus* NarG enzymes relative to the Proteobacteria. Together, these experiments begin to test which amino acid residues affect the stable isotopic signatures of the NarG enzyme, contributing to our understanding of the enzymatic controls of stable isotope effects.

**Fig. 1.**
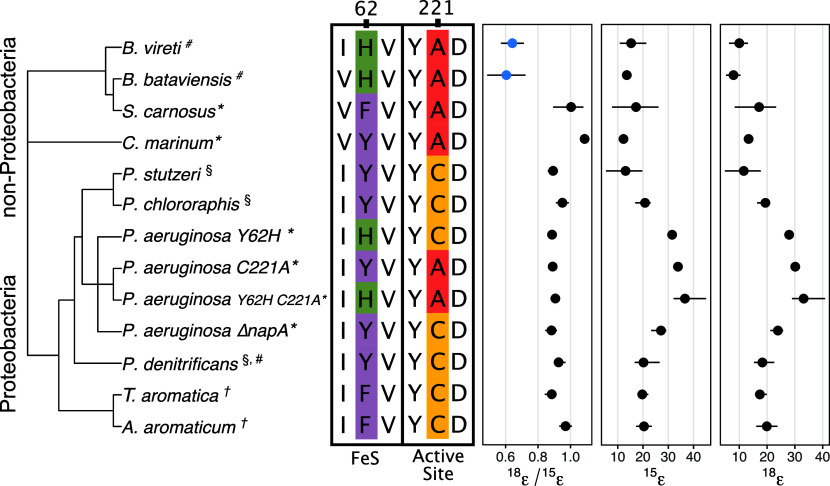
(*Left*) NarG maximum clade credibility phylogenetic tree. Isotopic data collected in this study denoted with an asterisk (*). Literature data indicated with (#)^4^, (†)^7^, (§)^2^. (*Middle*) NarG amino acid sequence alignment. In the *P. aeruginosa* NarG mutants constructed for this study, a histidine (H) was substituted for a tyrosine (Y) at position 62, and an alanine (A) was substituted for a cysteine (C). Position 222 is the aspartic acid (D) that coordinates the Mo atom. (*Right*) Distribution of ^18^ε/^15^ε, ^15^ε, and ^18^ε values. Blue datapoints in ^18^ε/^15^ε are the anomalous isotope signatures for NarG. Error bars show the maximum and minimum isotope values measured.

**Fig. 2.**
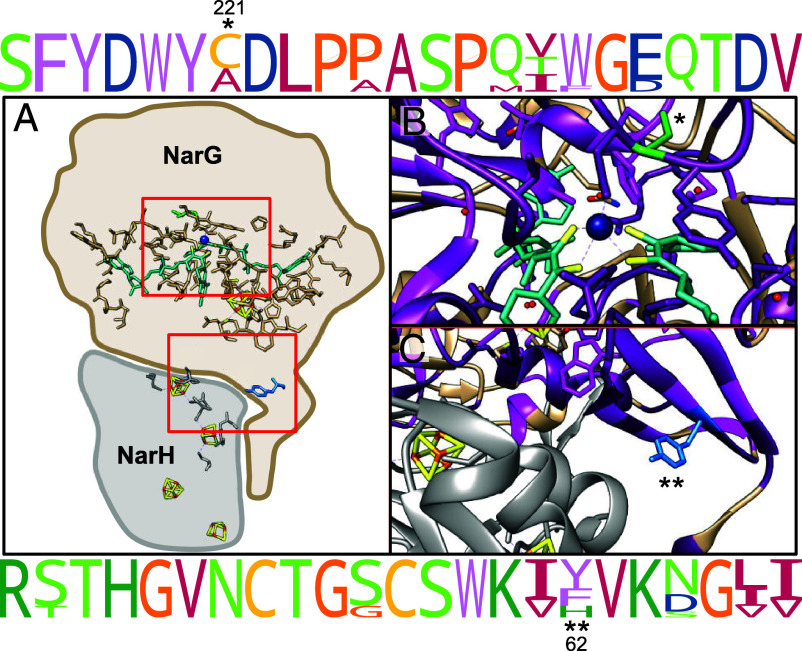
(*A*) The positions of residues C221 and Y62 in *E. coli’s* NarG structure (tan; cartoon not to scale). The molybdopterin guanine dinucleotide cofactor is colored teal, with coordinating sulfur atoms in yellow; PDB: 1Q16. (*B*) Closeup of position C221, colored green. The aspartic acid (D222) that coordinates the Mo atom (dark blue) is directly to the left of C221. (c) Y62, colored blue, is near FeS0 and is at the interface of NarH and NarG. Dark magenta ribbons indicate identical amino acid sequences across species. Light magenta ribbons indicate positions where amino acids are substituted with another residue with similar physiochemical properties as defined by the Zappo color scheme. (*Top*/*Bottom*) Consensus logos show the amino acid residues surrounding the mutated amino acids (indicated by * for C221 and ** for Y62). Consensus logos and ribbon coloration based on an alignment of NarG enzymes with published ^18^ε/^15^ε values.

## Results & Discussion

Under anoxic conditions in the presence of 25 mM NaNO_3_, the PA14 mutant strains Y62H and C221A grew to a similar density as the parent PA14 Δ*napA* strain (OD600 ~0.9), suggesting that the mutations did not impact overall growth yields. However, the mutant strain Y62H had a slower growth rate (0.29 ± 0.02 h^−1^) relative to those of the parent strain and the C221A mutant (0.52 ± 0.04 and 0.49 ± 0.03 h^−1^, respectively). The double mutant had a more variable and slightly faster growth rate of 0.67 ± 0.16 h^−1^ (within error of the parent).

C221A, Y62H, and the double mutant all displayed stronger isotope fractionation, with ^15^ε values of 33.8 ± 0.6‰, 31 ± 1.3‰, and 36.6 ± 7.3‰, respectively, versus the parent strain’s 27.1 ± 2.8‰ (Fig. 1). Despite these changes in the magnitude of fractionation, all mutants displayed near uniform ^18^ε/^15^ε of 0.89 ± 0.01 (Fig. 1). This suggests that either different or more substantial enzyme sequence changes are required to alter ^18^ε/^15^ε. This is noteworthy given that both of the mutations are within highly conserved regions of the NarG enzyme, with C221 in particular located directly next to the active site (Fig. 2*C* ). Furthermore, *S. carnosus* and *C. marinum* also exhibited typical NarG ^18^ε/^15^ε proportionalities of 0.93 ± 0.03 and 1.08 ± 0.02, respectively (Fig. 1), despite sharing many of the sequence changes observed in the two Bacilli.

These results indicate that ^18^ε/^15^ε signatures are more robust than anticipated, with amino acid residues beyond the active site likely involved in modulating the intrinsic isotope effect of NarG. While mutations around the active site are assumed to be most likely to influence ^18^ε/^15^ε, there are several remaining possibilities for what may control ^18^ε/^15^ε including direct or indirect oxygen exchange between the nitrate pool and water, unexpected changes in reversibility, and/or a yet unrecognized step in the pathway that influences the overall fractionation. We have previously tested the potential for oxygen exchange occurring in the *Bacillus* NarG enzyme using ^18^O labeled water and found no oxygen from water in the nitrate pool (4), suggesting that at least the first of these possibilities is an unlikely influence on ^18^ε/^15^ε.

Consequently, we turn our attention to other steps in the NR pathway that may impact the reaction’s rate limiting step and reversibility, such as electron transfer steps occurring within the NarI or NarH subunits. NarI collects electrons from the inner membrane and then transfers these electrons to NarH, which passes on the electrons to the catalytic subunit NarG (10). If nitrate binds molybedenum (Mo) in an oxidized state, the Mo atom must first revert to a reduced state before proceeding with NR (11). As discussed in Frey *et al.* 2014, this pause in NarG mediated NR may introduce an intramolecular isotope effect that contributes to the enzyme’s ^18^ε/^15^ε of ~0.9 (6). In contrast, during NapA-based NR, it is proposed that nitrate displaces one of the Mo-coordinating cysteine residues, binds directly to Mo, and then is reduced to nitrite using external reductants, *i.e.,* does not rely on internal electron transfer (11). This and NapA’s high nitrate affinity (10, 12) may limit the potential for an intramolecular isotope effect, thereby resulting in ^18^ε/^15^ε that is distinct from NarG. Future work addressing amino acid changes beyond the active site of the NarG enzyme, including amino acids near electron transferring FeS clusters or in other areas, such as the substrate tunnel, which may influence nitrate’s interaction with the active site, will be important to identify the true root of the ^18^ε/^15^ε signature.

Additionally, the results from *C. marinum* and *S. carnosus* suggest that the atypical NarG ^18^ε/^15^ε observed in *Bacillus* are potentially limited to the NarG variant found in this genus. This in turn would support the hypothesis that low ^18^ε/^15^ε (~0.6) found in freshwater and terrestrial ecosystems may stem from a predominance of NapA-based NR (2–4). As a counterpoint, the *Bacillus* genus is widespread across many ecosystems including freshwater and terrestrial environments(13) and its isotopically atypical NarG variant could contribute more to nitrogen cycling than previously anticipated. In either case, the co-occurrence of NR and nitrite reoxidation may also contribute to low ^18^ε/^15^ε ratios observed in freshwater and terrestrial ecosystems (1).

Along with evaluating a genetic basis for the ^18^ε/^15^ε signature, the *Bacillus* NarG anomaly may offer insight into the fidelity of stable isotopic signatures through time. It is conceivable that enzymes collect mutations through time that change their intrinsic isotopic signatures significantly such that the signatures preserved in the rock record do not reflect their modern equivalents. However, the degree to which enzyme mutations could potentially influence isotopic signatures remains largely untested. While stable isotopes of nitrate are not commonly preserved in the rock record, other enzymatically produced signatures, such as those from carbon fixation (14) and sulfate reduction (15), are. Importantly, while bacterial growth rates appear to impact the magnitude of nitrate isotopic effects, previous work shows that the ^18^ε/^15^ε proportionality of dissimilatory NR is conserved regardless of the magnitude of isotope fractionation (13). This makes the ^18^ε/^15^ε proportionality a powerful model system for identifying changes to isotopic phenotypes without uncertainties from variability in bacterial growth rates or the degree to which the isotopic fractionations are expressed at the environmental scale. The findings of this work may thus guide future studies aimed at testing the resilience of stable isotopic signatures through time.

## Conclusions

Our data indicate that the root of the ^18^ε/^15^ε signature created by the NarG enzyme may be related to amino acid residues beyond the active site. However, a precise mechanism for the formation of this stable isotope signature still remains elusive. This study also adds to the small body of work that tests the uniformity of stable isotopic signatures through time through directed mutations and artificial evolution experiments (14, 16, 17). Our data tentatively support the hypothesis that stable isotope signatures are more evolutionarily robust than anticipated. This matches previous reports that modern isotopic signatures can accurately represent their ancient counterparts (14). It is important to keep in mind, however, that this study investigated a limited number of mutations in a single enzyme, and future work is required to gain a more complete understanding of how isotopic phenotypes vary in response to evolutionary change.

## Materials and Methods

Experiments and isotopic analyses were run as in Asamoto *et al.* (2021)(4). Briefly, batch experiments were conducted anaerobically in triplicate in sealed Balch tubes. Nitrate samples were collected as bacteria consumed nitrate. Stable isotopic data were collected using the denitrifier method (18). See SI Appendix for details about the bacterial strains, culturing conditions, mutant strain construction, isotope calculations, ion chromatography parameters, NCBI accession numbers for NarG sequences, and methods used for the NarG sequence alignment. All data and source code used to analyze and produce the figures for this paper are available at (19).

## Supplementary Material

Appendix 01 (PDF)

## Data Availability

Isotopic, culture, and nitrate concentration data have been deposited in Zenodo (19).

## References

[1] J. Granger, S. D. Wankel, Isotopic overprinting of nitrification on denitrification as a ubiquitous and unifying feature of environmental nitrogen cycling. Proc. Natl. Acad. Sci. U.S.A. **113**, E6391–E6400 (2016). 27702902 10.1073/pnas.1601383113 PMC5081596

[2] J. Granger, D. M. Sigman, M. F. Lehmann, P. D. Tortell, Nitrogen and oxygen isotope fractionation during dissimilatory nitrate reduction by denitrifying bacteria. Limnol. Oceanogr. **53**, 2533–2545 (2008).

[3] L. A. Treibergs, J. Granger, Enzyme level N and O isotope effects of assimilatory and dissimilatory nitrate reduction. Limnol. Oceanogr. **62**, 272–288 (2017).

[4] C. K. Asamoto, K. R. Rempfert, V. H. Luu, A. D. Younkin, S. K. Kopf, Enzyme-specific coupling of oxygen and nitrogen isotope fractionation of the Nap and Nar nitrate reductases. Environ. Sci. Technol. **55**, 5537–5546 (2021). 33687201 10.1021/acs.est.0c07816

[5] K. Kritee , Reduced isotope fractionation by denitrification under conditions relevant to the ocean. Geochim. Cosmochim. Acta **92**, 243–259 (2012).

[6] C. Frey, S. Hietanen, K. Jürgens, M. Labrenz, M. N. Voss, O Isotope fractionation in nitrate during chemolithoautotrophic denitrification by *Sulfurimonas gotlandica*. Environ. Sci. Technol. **48**, 13229–13237 (2014). 25347214 10.1021/es503456g

[7] A. Wunderlich, R. Meckenstock, F. Einsiedl, Effect of different carbon substrates on nitrate stable isotope fractionation during microbial denitrification. Environ. Sci. Technol. **46**, 4861–4868 (2012). 22458947 10.1021/es204075b

[8] Y. He, Y. Zhang, S. Zhang, Y. Liu, Predicting nitrogen and oxygen kinetic isotope effects of nitrate reduction by periplasmic dissimilatory nitrate reductase. Geochim. Cosmochim. Acta **293**, 224–239 (2021).

[9] W. Guo, J. Granger, D. M. Sigman Nitrate Isotope Fractionations During Biological Nitrate Reduction: Insights from First Principles Theoretical Modeling (AGU Fall Meeting, 2010).

[10] D. J. Richardson, B. C. Berks, D. A. Russell, S. Spiro, C. J. Taylor, Functional, biochemical and genetic diversity of prokaryotic nitrate reductases: CMLS. Cell. Mol. Life Sci. **58**, 165–178 (2001). 11289299 10.1007/PL00000845 PMC11146511

[11] C. Coelho, M. J. Romão, Structural and mechanistic insights on nitrate reductases. Protein Sci. **24**, 1901–1911 (2015). 26362109 10.1002/pro.2801 PMC4815237

[12] C. Sparacino-Watkins, J. F. Stolz, P. Basu, Nitrate and periplasmic nitrate reductases. Chem. Soc. Rev. **43**, 676–706 (2014). 24141308 10.1039/c3cs60249d PMC4080430

[13] I. Mandic-Mulec, P. Stefanic, J. D. van Elsas, Ecology of Bacillaceae. Microbiol. Spectrum **3**, (2015), 10.1128/microbiolspec.tbs-0017-2013. 26104706

[14] M. Kędzior , Resurrected Rubisco suggests uniform carbon isotope signatures over geologic time. Cell Rep. **39**, 110726 (2022). 35476992 10.1016/j.celrep.2022.110726

[15] B. A. Wing, I. Halevy, Intracellular metabolite levels shape sulfur isotope fractionation during microbial sulfate respiration. Proc. Natl. Acad. Sci. U.S.A. **111**, 18116–18125 (2014). 25362045 10.1073/pnas.1407502111 PMC4280625

[16] A. Pellerin , Sulfur isotope fractionation during the evolutionary adaptation of a sulfate-reducing bacterium. Appl. Environ. Microbiol. **81**, 2676–2689 (2015). 25662968 10.1128/AEM.03476-14 PMC4375314

[17] S. J. Hurley, B. A. Wing, C. E. Jasper, N. C. Hill, J. C. Cameron, Carbon isotope evidence for the global physiology of Proterozoic cyanobacteria. Sci. Adv. **7**, eabc8998 (2021). 33523966 10.1126/sciadv.abc8998 PMC7787495

[18] M. A. Weigand, J. Foriel, B. Barnett, S. Oleynik, D. M. Sigman, Updates to instrumentation and protocols for isotopic analysis of nitrate by the denitrifier method: Denitrifier method protocols and instrumentation updates. Rapid. Commun. Mass Spectrom. **30**, 1365–1383 (2016). 27197029 10.1002/rcm.7570

[19] C. K. Asamoto , Data. Zenodo. 10.5281/zenodo.13259971. Deposited 7 August 2024.

