# Supplementary material for: Stable isotopic signature of dissimilatory nitrate reduction is robust against enzyme mutation
Source: Proc Natl Acad Sci U S A. 2024 Nov 22;121(48):e2416002121. doi: 10.1073/pnas.2416002121 (PMC11621745; doi:10.1073/pnas.2416002121)
Supplement: Supplementary file 1 — Appendix 01 (PDF) [file pnas.2416002121.sapp.pdf]

PNAS

Supporting Information for

Stable isotopic signature of dissimilatory nitrate reduction is robust against enzyme mutation.

Ciara K. Asamoto, Yeongjun Ryu, Kelly N. Eckartt, Julia Kelley-Kern, Lars E.P. Dietrich, Daniel M. Sigman, Sebastian H. Kopf.

Ciara K. Asamoto
Email: ciara.asamoto@colorado.edu

This PDF file includes:

Supporting text
SI References

Supporting Information Text

Extended Methods:

Strain information and culturing conditions

Staphylococcus carnosus (DSM 20501) was cultured in a modified basal medium at 37°C [1]. In g/L it contained 5g of yeast extract, 13g K_2HPO_4, 2g NaCl and 2.1g $NaNO_3$ was added. *Corynebacterium marinum* (DSM 44953) was cultured at 30°C in 15g/L Trypticase Soy Broth (TSB) with 2% NaCl and 25mM $NaNO_3$ added. The *Pseudomonas aeruginosa* mutant strains were cultured in LB medium with 25mM $NaNO_3$ added. The media for all strains was sparged with N_2 gas and dispensed into balch tubes. Filter sterilized sodium lactate was added to the *S. carnosus* media post autoclave to a final concentration of 25mM.

PA14 mutant construction

The PA14 mutant was constructed in a PA14 strain with *napA* removed from its genome (PA14 Δ*napA*) to avoid any interfering isotopic signal from NapA [2]. Approximately 500 base pairs each were amplified for the upstream and downstream regions of the targeted amino acid within the *narG* gene. The desired mutation was made by inserting the *Bacillus*-specific codon into the center of the reverse primer of the upstream region with 10 complementing base pairs before and after the mutation. The reverse complement of the reverse primer was used as the forward primer for the downstream region. Yeast gap repair cloning was done with yeast strain InvSc1, a URA3 mutant, to assemble the up- and downstream regions into a pMQ30 plasmid that had been digested with BamHI and EcoRI. The pMQ30 plasmid contains the yeast URA3 gene for uracil production, a *sacB* gene for sucrose selection, and gentamicin-resistance cassette. Yeast that correctly assembled the plasmid were selected on synthetic defined plates without uracil. The assembled plasmid was transformed into chemically competent *E. coli* (UQ950) cells to increase plasmid yields. Successful transformants were conferred gentamicin resistance and selected for on LB plates containing gentamicin. Purified plasmid was transformed into the mating strain BW29427 and then transferred to PA14 Δ*napA* via conjugation. PA14 single recombinants were selected on LB agar plates containing 100 µg/ mL gentamicin. Double recombinants (markerless substitutions) were selected on LB without NaCl and modified to contain 10% sucrose. To make the double-mutant strain (PA14 Δ*napA* Y62H C221A), the pMQ30 plasmid harboring the Y62H mutant was conjugated into PA14 Δ*napA* C221A. Genotypes of substitution mutants were confirmed by PCR.

Batch Experiments

All strains were passaged between 3-5 times in aerobic culture before being inoculated into anaerobic balch tubes. Each experiment had three biological replicates that were sampled as the strains consumed nitrate. OD_{630} measurements were collected continuously for the *S. carnosus* experiment. OD_{600} was taken at each sample timepoint for PA14 mutant experiments. Sequencing over the narG active site was done at the end of the batch experiments with PA14 ΔnapA SDM to ensure the mutation was present in all replicates. Isotopic samples were filter sterilized with a 0.2µm PES filter into an acid washed tube and immediately frozen at -20°C. A fraction of this sample was diluted into water for ion chromatography analysis. 0.1M NaOH was added to stabilize nitrite at a final pH of 11 and then stored at -20°C.

Ion Chromatography

Nitrate and nitrite concentrations were quantified using a Dionex ICS-6000 Ion Chromatograph equipped with an IonPac AS11-HC column and a variable wavelength absorbance (UV/Vis) detector. Samples were eluted isocratically with 25mM KOH at a flow rate of 1.5mL/ minute. Nitrate and nitrite peaks were measured at a wavelength of 210nm and quantified against laboratory standards. Nitrate from the samples were purified via fraction collection and re-analyzed to ensure there was no nitrite carry over. If any residual nitrite was measured, nitrite removal was performed prior to isotopic analysis using the sulfamic acid method [3]. The error for IC measurements was calculated based on the residual errors of the standard curve during each IC run. The nitrate and nitrite standards used for calibration ranged between 5 – 250µM.

Isotope Analysis

The N and O isotopic composition of nitrate was determined using the denitrifier method [4,5] using *Pseudomonas aureofaciens* with 20 nmol nitrate per analysis. The isotopic measurements were calibrated against the potassium nitrate reference standards IAEA-NO3 ($\delta^{15}N$ = 4.7‰ vs. air, $\delta^{18}O$ = 25.6‰ vs. Vienna Standard Mean Ocean Water (VSMOW)), provided by the International Atomic Energy Agency and USGS34 ($\delta^{15}N$ = -1.8‰ vs. air, $\delta^{18}O$ = -27.9‰ vs. VSMOW) provided by the United States Geological Survey, each measured at two different concentrations every 8 samples to correct for injection volumes. Analytical runs were corrected for instrument drift based on an N_2O drift monitoring standard. All isotopic data are reported in conventional delta notation versus the international reference scales for N (Air) and O (VSMOW): $\delta^{15}N = ([^{15}N/^{14}N]_{sample}/[^{15}N/^{14}N]_{air} - 1)$ and $\delta^{18}O = ([^{18}O/^{16}O]_{sample}/[^{18}O/^{16}O]_{VSMOW} - 1)$. δ values reported in per mil (‰) are implicitly multiplied by a factor of 1000 [6]. A technical replicate for all timepoints for *C. marinum* replicate 1 was also analyzed and labeled as replicate 4 in the data.

The nitrate $\delta^{15}N$ and $\delta^{18}O$ measurements were fit to the following linear equations to estimate the N and O isotope effects ($^{15}\varepsilon$ and $^{18}\varepsilon$) and isotope effect proportionality ($^{18}\varepsilon$ / $^{15}\varepsilon$) imparted on nitrate during microbial nitrate reduction from the slope of the regressions [7]:

$$\ln\left(\frac{\delta^{18}O + 1}{\delta^{18}O_{initial} + 1}\right) = -{}^{18}\varepsilon \cdot \ln(f) = \frac{{}^{18}\varepsilon}{{}^{15}\varepsilon} \cdot \ln\left(\frac{\delta^{15}N + 1}{\delta^{15}N_{initial} + 1}\right) = -{}^{15}\varepsilon \cdot \ln(f)$$

where $f = [NO_3^-]/[NO_3^-]_{initial}$ is the fraction of nitrate remaining and δ and ε values in per mil (‰) are implicitly multiplied by a factor of 1000 [6]. The errors of the regression slopes were used to estimate standard errors for $^{15}\varepsilon$ (eq 1), $^{18}\varepsilon$ (eq 2), and $^{18}\varepsilon$ / $^{15}\varepsilon$ (eq 3). Note that for this implementation of the Rayleigh distillation model, normal kinetic isotope effects (reflecting higher reaction rates of the lighter isotopes) are positive ($\varepsilon > 0$), which is a common convention in the nitrate reduction literature and thus used in this publication. We point this out explicitly because readers more familiar with other isotope systems (e.g. C and H) where normal kinetic isotope effects are typically reported as negative numbers ($\varepsilon < 0$) may find this convention counter-intuitive.

Protein Sequence Analysis

The NarG sequences were aligned using Clustal Omega Multiple Sequence Alignment. Maximum clade credibility NarG protein trees were constructed used MrBayes' Markov chain Monte Carlo analysis under fixed rate amino acid model with default parameters [8]. *E. coli* protein structure (PDB: 1Q16) was used as a reference for a typical NarG structure [9].

NCBI Accession Numbers for NarG sequences used in alignment

Strain	NCBI Accession Number
Staphylococcus carnosus	KOR12931.1
Pseudomonas stutzeri	ABP78593.1
Pseudomonas aeruginosa PA14	EOT11604
Aromatoleum aromaticum EbN1	WP_011239378.1
Paracoccus denitrificans PD1222	WP_011750465.1
Bacillus bataviensis LMG 21833	EKN65800.1
Bacillus vireti LMG 21834	ETI68959.1
Pseudomonas chlororaphis subsp chlororaphis ATCC 9446	AIC20663.1
Thauera aromatica K172	AVR88636.1
Corynebacterium marinum	AJK68610.1

SI References

1. Neubauer, H. & Götz, F. Physiology and interaction of nitrate and nitrite reduction in Staphylococcus carnosus. *J Bacteriol* **178**, 2005–2009 (1996).
2. Dietrich, L. E. P. *et al.* Bacterial Community Morphogenesis Is Intimately Linked to the Intracellular Redox State. *Journal of Bacteriology* **195**, 1371–1380 (2013).
3. Granger, J. & Sigman, D. M. Removal of nitrite with sulfamic acid for nitrate N and O isotope analysis with the denitrifier method. *Rapid Commun. Mass Spectrom.* **23**, 3753–3762 (2009).
4. Sigman, D. M. *et al.* A Bacterial Method for the Nitrogen Isotopic Analysis of Nitrate in Seawater and Freshwater. *Anal. Chem.* **73**, 4145–4153 (2001).
5. Weigand, M. A., Foriel, J., Barnett, B., Oleynik, S. & Sigman, D. M. Updates to instrumentation and protocols for isotopic analysis of nitrate by the denitrifier method: Denitrifier method protocols and instrumentation updates. *Rapid Commun. Mass Spectrom.* **30**, 1365–1383 (2016).
6. Coplen, T. B. Guidelines and recommended terms for expression of stable-isotope-ratio and gas-ratio measurement results: Guidelines and recommended terms for expressing stable isotope results. *Rapid Commun. Mass Spectrom.* **25**, 2538–2560 (2011).
7. Mariotti, A. *et al.* Experimental determination of nitrogen kinetic isotope fractionation: Some principles; illustration for the denitrification and nitrification processes. *Plant Soil* **62**, 413–430 (1981).
8. Huelsenbeck, J. P. & Ronquist, F. MRBAYES: Bayesian inference of phylogenetic trees. *Bioinformatics* **17**, 754–755 (2001).
9. Bertero, M. G. *et al.* Insights into the respiratory electron transfer pathway from the structure of nitrate reductase A. *Nat Struct Mol Biol* **10**, 681–687 (2003).
